# Relationship between family meal frequency and individual dietary intake among diabetic patients

**DOI:** 10.1186/s40200-015-0187-5

**Published:** 2015-08-08

**Authors:** Divya Ruhee, Fawzi Mahomoodally

**Affiliations:** Department of Health Sciences, Faculty of Science, University of Mauritius, 230 Réduit, Mauritius

**Keywords:** Diabetes, Individual dietary intake, Family meals, Family cohesion, Mediation analysis

## Abstract

**Background:**

Notoriously, the island of Mauritius has one of the highest prevalence of diabetes in the world. Management of the disease is very important and family meals are undoubtedly beneficial to patients as they promote the development of healthy eating behaviours and food choices. This study has aimed to probe into potential relationship(s) between family meal frequency and individual dietary intake among diabetic patients and to establish whether family cohesion may be a plausible mediator of this relationship.

**Methods:**

A cross-sectional survey was carried out with a random sample of 384 diabetic patients. The Family Adaptability and Cohesion Evaluation Scale III was used to obtain information on two general aspects of family functioning, that is, cohesiveness and adaptability. Chi-squared (*χ*^2^) tests, independent sample *t*-tests and one-way ANOVA were used to determine statistical significance. Pearson correlation was used to examine associations between family meal frequency, individual dietary intake and family cohesion. Hierarchical linear regression models were performed for the mediation analysis.

**Results:**

Family meal frequency (breakfast, lunch and dinner) was observed to be positively associated with intake of fish, raw vegetables, dried and fresh fruits, low-fat milk, cheese, yogurt, nuts and light butter and negatively associated with intake of red meat, white rice, white bread, whole egg fried, chocolates, fried cakes, burgers, chips, and fried noodles/rice. Average mediation (52.6 %) was indicated by family cohesion for the association between family meal frequency and individual dietary intake among diabetic patients. Sobel’s test further confirmed the trend towards complete mediation (z = 15.4; *P* < 0.05).

**Conclusions:**

A strong relationship between family meal frequency and individual dietary intake among diabetic patients was recorded. The present study is one of the few studies that have examined family cohesion as a mediator of the relationship and to our best knowledge is the first work to demonstrate a trend towards complete mediation. Results obtained can be used by health professionals to devise strategies for increasing knowledge and awareness of both diabetic patients and their respective families to curd down this public health burden.

## Background

The World Health Organisation (WHO) defines diabetes as a chronic metabolic disease that occurs either when the pancreas does not produce enough insulin or when the body cannot effectively use the insulin it produces [33]. Diabetes is unfortunately one of the biggest public health challenges in human history and it is affecting even more people around the world each year. Even though the real aetiology of diabetes is not fully understood by experts, the associated risk factors are numerous ranging from genetic triggers to environmental ones. It becomes imperative to detect and manage the disease as soon as possible because excessive fluctuations in blood glucose level can injure tissues throughout the whole body [31]. Most importantly there is strong evidence that the disease is the leading cause of cardiovascular diseases, renal problems, limb amputation, gangrene, peripheral nerve damage and visual impairment [2, 28].

About 382 million people, in 2013, have diabetes in the world and it is expected to rise up to 592 million by 2035 if serious attention is not drawn towards the problem [14]. Mauritius is one of the countries with very high prevalence rate of diabetes and currently about 290,000 people suffer from diabetes with various health complications such as hypertension, renal failure and visual disorders [22]. Along with being a worrying situation in Mauritius, diabetes mellitus is equally associated with costs in terms of human suffering, health care and loss resources [22]. Hence, effective treatment for diabetes is of uttermost importance.

Pharmacotherapy is normally used by most diabetic patients as the primary means of achieving optimal glycaemic control. However, most drugs are usually associated with weight gain. Therefore, medical nutrition therapy (MNT) and lifestyle intervention remains the cornerstone for the management of diabetes, especially those with NIDDM [18]. Along with diet and exercise, the role of family functioning as a factor in the management of the disease has been suggested in many studies [26]. Various aspects of the family meal environment such as characteristics of the family during mealtimes, frequency of family meals and the atmosphere play a crucial part in modelling food-related behaviours [3].

Previous studies have focused mostly on the influence of family meals on the consumption of fruits and vegetables in adolescents. Moreover very few studies have been conducted on the relationship between family meal frequency and individual dietary intake and therefore there is no established theory to explain the association among diabetic patients in Mauritius. Family cohesion, defined as the emotional bonding that family members have towards one another, may have significant implications for the adoption of healthful eating habits [32]. It can therefore be hypothesised that the frequency of family meals reflects the family social environment and family cohesion, which may influence the individual dietary intake among diabetic patients. In the light of previous findings, it can also be hypothesised that greater frequency of family meals is associated with higher fruits and vegetables intake and lower consumption of high-calorie snacks, sweets and sweetened drinks among diabetic patients [3, 33]. Henceforth, from all these studies and analyses, it can be said that there may be a relationship between family meals and family cohesion that had a direct impact on individual dietary intake. However, this relationship is not clear and need further investigation. Explaining the mechanism through which family meal frequency has a positive influence on individual dietary intake with cohesion as a possible mediator can help to intensify dietary interventions to improve the situation in Mauritius. The purpose of this project is therefore to determine whether there is a relationship between family meal frequency and individual dietary intake among diabetic patients in Mauritius and to examine family cohesion as a possible mediator of this relationship.

## Methods

### Design

A self-administered cross-sectional survey was conducted to meet the objectives of the study since it is a quantitative data collection approach targeted to obtain information from a representative sample of the population. For a population of about 290,000 diabetic patients in Mauritius, the requested sample size, for ± precision with 95 % confidence interval (CI) and degree of variability, p equal to 0.5, was found to be 384. To make the sample representative of the population suffering from diabetes mellitus, participants were recruited randomly from health care centres all around the island from both rural and urban areas. Non-governmental organizations such as Mauritius Diabetes Association, T1diams, Diabetes Parent Support Group and ‘Groupement des Diabétiques’ were also targeted.

The respondents selected for the study had to meet the following inclusion criteria: (1) Type I and Type II diabetic patients only were recruited; (2) patients above 12 years; (3) Participants who are mentally fit to answer questions; and (4) Participants should be of Mauritian origin and they should be able to understand English, French and Creole (vernacular) language. The exclusion criteria included patients suffering from other types of diabetes, except Type I and Type II. Diabetic patients suffering from extremely poor health conditions and having difficulties to understand and answer questions were also excluded.

### Design and content of questionnaire

The questionnaire comprised of 20 main questions and divided into four distinct sections; personal data such as socio-demographic information; individual dietary intake; family meal frequency and family support and cohesion [13].

Section A consisted of questions based on personal and socio-demographic information such as age, sex, ethnic group, residential area, educational background, occupation, income group, marital status and number of persons in the family which are considered to be predictors of self care behaviour. Questions on types of diabetes, family history of diabetes and body mass index (BMI) were also being asked. BMI was calculated as weight (kg)/height squared (m^2^). The weight of participants, with their shoes removed, was recorded by the first author using a calibrated digital scale and the height was measured using a measuring tape.

Section B consisted of questions based on individual dietary intake such as vegetarianism, number of meals taken each day, skipping meals and glycaemic index. Dietary intake of diabetic patients was assessed using a self-administered food frequency questionnaire adapted from the Diet History Questionnaire [32]. In this study, the food frequency questionnaire was modified and adapted. For instance, the questionnaire was translated to French and to the local vernacular language ‘creole’. Prior to start of the full-scale survey, a pilot testing was conducted with ten subjects, in order to judge the clarity of the questions. A few amendments were then brought to the questionnaire, where some of the questions were rephrased due to ambiguity. Response options consisted of 5 categories such as never, 1–2 times, 3–4 times, 5–6 times and everyday and they were recorded to reflect an average frequency of consumption per week such as 0, 1.5, 3.5, 5.5 and 7 times per week. In addition, participants were also asked to report their average serving size of consumed items which consisted of four categories of response options such as ½ cup, 1 cup, 2 cups and more than 2 cups and average sizes were recorded as 0.5, 1, 1.5 and 2 servings respectively. The average number of servings per week of given items was calculated by multiplying frequency of consumption by average servings [32].

Section C was based on family meal frequency which was assessed by the question, “In the past week, how many times did you and your family (living in your house) had breakfast, lunch and dinner together?” Responses were accepted as never, 1–2 times, 3–4 times and every day.

Section D consisted of questions on family support and cohesion. The Family Adaptability and Cohesion Evaluation Scale III (FACES III) is a validated 20-item self-reporting scale which was used to obtain information on two general aspects of family functioning, that is, cohesiveness and adaptability [12 http://www.eruralfamilies.org]. Family cohesion assesses degree of separation or connection of family members to the family. There are four levels of family cohesion ranging from extreme low cohesion to extreme high cohesion- disengaged, separated, connected, and enmeshed. There are four levels of adaptability - rigid, structured, flexible, and chaotic [21]. Through various studies conducted, it became well known that cohesiveness is a more predictive and powerful variable than adaptability [21]. Therefore only the cohesion dimension was used in the analysis of the study. The cohesion scale of FACES-III (see Table 1) contained 10 items. Participants were asked to describe their family or household as it was by checking the appropriate number in the boxes for each item. All the responses were recorded on a five-point scale ranging from 1 (“almost never”) to 5 (“almost always”). Therefore the lowest possible score is 10 and the maximum score is 50, and the higher the score, the greater perceived family cohesion [21].

**Table 1:** Characteristics and interpretation of the Family Adaptability and Cohesion Evaluation Scale III

Subscale	Scoring range	Interpretation
Cohesion scale	10-50	10-34 : Disengaged family
		35–40 : Separated family
		41–45 : Connected family
		46–50 : Enmeshed family

### Data collection

The questionnaire was designed in English language and translated in Creole, a commonly spoken local language in Mauritius, or French for those who were not comfortable with English language. However, in cases where participants had language and communication issues, data was collected using face to face interviews where the questionnaire was filled out by the first author. Participation to the study was voluntary and formal consent was obtained from all the respondents. An informed consent form was provided at the beginning of each questionnaire where they were assured of the confidentiality of the data collected [7, 17].

### Statistical analysis of data

**Fig. 1:**
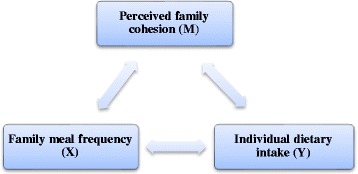
Associations between the different variables in the study

Data was analysed using SPSS (version 16.0) software and Excel 2007. Chi squared (*χ*^2^) test, independent sample t-tests and one-way ANOVA analysis were performed to compare the various socio-demographic variables, family cohesion scores and eating behaviours of diabetic patients. Pearson correlation coefficients were used to examine the associations between family cohesion and family meal frequency. The Baron and Kenny approach as described by Welsh et al. [32] was the main statistical method used in this study. There were four steps that were adopted to prove the presence of a mediator, that is, family cohesion:

1. To demonstrate that the independent variable X (family meal frequency) was associated with the outcome variable Y (individual dietary intake).
2. To demonstrate that the independent variable X was associated with the mediator variable M (family cohesion).
3. To demonstrate that the mediator variable M was associated with the outcome variable Y after controlling for X.
4. The last step consisted of determining whether the effect of X on Y was reduced when M was included in a regression model, providing evidence that the effect of X on Y was partially mediated by M. Furthermore, if the path between X and Y disappeared completely when M was included, complete mediation by M was indicated [see Fig. 1].

A series of hierarchical multiple linear regression models were performed where each diabetic patient’s average weekly servings of a targeted food or beverage item were regressed on frequency of family meals and included a random effect for household (family cohesion). Similar regression models were fit regressing family cohesion scores on frequency of family meals as well as regressing average servings of targeted food or beverage items on family cohesion scores after controlling for frequency of family meals. Regression models regressing weekly servings of a targeted food category on both family meal frequency and family cohesion score were fit in order to provide evidence for mediation. When mediation was indicated, the percentage reduction for the association of family meals and individual dietary intake as mediated by family cohesion was calculated by subtracting the adjusted regression coefficient for family meal frequency from its unadjusted counterpart and then dividing by the unadjusted regression coefficient for frequency of family meals [32]. For each of those tests, a *P* value of less than 0.05 was considered as statistically significant.

## Results

### Demographic profile

A total of 384 participants completed the questionnaire which was representative of the Mauritian population. Socio-demographic variables such as gender, age group, ethnic group, demographic area, marital status, educational background, occupation, income group, number of persons in family, type of diabetes and family history of diabetes are displayed in Table 2.

**Table 2:** Descriptive statistics for socio-demographic variables

Socio-demographic Variables	n	%
Gender		
Male	208	54.2
Female	176	45.8
Age group		
12-17	44	11.5
18-30	59	15.4
30-60	208	54.2
>60	73	19.0
Ethnic group		
Christian	102	26.6
Muslim	65	16.9
Hindu	187	48.7
Chinese	28	7.3
General Population	2	0.5
Demographic area		
Urban	238	62.0
Rural	146	38.0
Marital status		
Married	234	60.9
Single	150	39.1
Educational background		
None	2	0.5
Primary	71	18.5
Secondary	190	49.5
Tertiary	121	31.5
Occupation		
Student	58	15.1
Manual Worker	42	10.9
Housewife/Unemployed	82	21.9
Office/managerial	147	38.2
Others	63	13.8
Income group		
<Rs.10,000	145	37.8
Rs.10,000-Rs. 20,000	161	41.9
>Rs.20,000	78	20.3
Number of persons in family		
1	8	2.1
2-3	154	40.1
4-6	168	43.8
More than 6	54	14.1
Type of Diabetes		
Type 1	82	21.4
Type 2	302	78.6
Family history of diabetes		
Yes	290	75.5
No	94	24.5
Member of family with diabetes		
Mother	82	21.4
Father	94	24.5
Mother and Father	65	16.9
Grandparents	49	12.8
N/A	94	24.5

### Anthropometric measurement

**Fig. 2:**
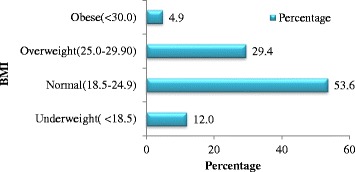
Percentage of the different categories of BMI

The BMI was calculated from the measured height and weight of the respondents and Fig. 2 illustrates the number of individuals, in the different BMI categories. As it can be seen 53.6 % of the respondents were normal with a BMI of “18.5-24.9” whereas the rest was either underweight or overweight or obese carrying a percentage of 12.0 %, 29.4 % and 4.9 % respectively which tend to corroborate with actual figures depicted by the growing number of overweight and obese people in Mauritius.

### Individual dietary intake

The eating behaviours and food consumption of the participants were assessed using different questions on vegetarianism, number of meals per day, skipping meals and knowledge of glycaemic index. The Food Frequency Questionnaire indicated the food intake of specific food items. It was found that 99.0 % of the subjects were non-vegetarian and only 1.0 % were vegetarian. 52.6 % consumed “3 meals” per day while the rest had “less than 3 meals”, “5-7 meals” and “more than 7 meals” with percentage occurrence of 9.4 %, 28.9 % and 9.1 % respectively (see Table 3). 60.7 % of the respondents do not skip meals whereas the remaining 39.3 % do skip meals.

**Table 3:** Descriptive statistics of eating behaviours of subjects

Eating behaviour	n	%
Vegetarianism		
Yes	4	1.0
No	380	99.0
Number of meals/day		
<3	202	52.6
3	36	9.4
5-7	111	28.9
More than 7	35	9.1
Skipping meals		
Yes	151	39.3
No	233	60.7
Glycaemic index		
Yes	10	2.6
No	374	97.4

It was observed that 97.4 % of the participants were ignorant about the meaning of “glycaemic index” and only 2.6 % were aware of it. Participant-reported food consumption of the different food items are shown in Table 4. Statistical comparisons of the mean servings per week of the food items demonstrated that the respondents had a higher consumption of rice (white), bread (white), raw vegetables, fresh fruits and water with mean values of 6.24, 6.33, 6.06, 6.26 and 7.25 respectively. Lower consumption was recorded for beef, pork, liver/organ meat, dholl-puri/farata, whole egg boiled, egg without yolk, root vegetables, dried fruits, chocolates, pastries, gateaux-piments, light butter, diet drinks and artificial sweeteners with mean values of 0.191, 0.110, 1.32, 1.63, 1.55, 1.13, 1.57, 1.82, 1.63, 0.93, 1.81, 1.99, 0.457 and 0.74 respectively.

**Table 4:** Average number of servings per week of the given food items from food frequency questionnaire

Food items	Mean ± SD
Meat products	
Chicken	5.95 ± 4.77
Lamb/mutton	3.44 ± 4.57
Beef	0.19 ± 0.69
Pork	0.11 ± 0.44
Liver/organ meat	1.32 ± 1.73
Fish	2.54 ± 2.44
Pulses	5.90 ± 2.94
Cereals	
Rice(white)	6.24 ± 4.31
Rice(brown)	0.45 ± 1.32
Bread(white)	6.33 ± 4.28
Bread(brown)	2.78 ± 3.35
Pasta	2.47 ± 3.19
Dholl-puri/farata	1.63 ± 3.11
Egg	
Whole egg boiled	1.55 ± 1.85
Whole egg fried	3.48 ± 4.47
Egg without yolk	1.13 ± 1.56
Tofu	3.31 ± 3.39
Soya	3.19 ± 3.46
Vegetables	
Raw	6.06 ± 5.58
Root	1.57 ± 1.56
Fruits	
Dried	1.82 ± 2.04
Fresh	6.26 ± 5.35
Dairy products	
Full-fat milk	3.62 ± 3.61
Low-fat milk	3.78 ± 3.52
Cheese	2.36 ± 2.40
Yogurt	3.21 ± 2.85
Snacks	
Chocolates	1.63 ± 2.25
Sweets	2.15 ± 2.44
Pastries	0.93 ± 1.47
Nuts	2.65 ± 3.06
Gateaux-piments	1.81 ± 2.13
Butter	
Ghee/saturated fats	3.04 ± 4.43
Light butter	1.99 ± 1.82
Drinks	
Soft drinks	2.95 ± 4.29
Water	7.25 ± 3.19
Diet drinks	0.46 ± 1.19
100 % fresh juice	2.42 ± 2.59
Fast foods	
Burger	2.71 ± 3.37
Chips	2.74 ± 3.36
Pizza	2.14 ± 3.31
Briani	2.84 ± 3.41
Fried noodles/rice	2.85 ± 3.39
Artificial sweeteners	0.74 ± 2.14

### Family meal frequency

The frequency of family meals was reported by participants in three categories namely “eat breakfast together”, “eat lunch together” and “eat dinner together” and summarised in Table 5. The percentage of respondents who never ate breakfast together as a family was 50.8 % and 56.2 % never ate lunch as a family.

**Table 5:** Participants’ reported frequency of family meals per week

Family meal frequency	n	%
Eat breakfast together		
Never	195	50.8
1-2 times	26	6.8
3-4 times	6	1.6
Everyday	157	40.9
Eat lunch together		
Never	216	56.2
1-2 times	14	3.6
3-4 times	46	12.0
Everyday	108	28.1
Eat dinner together		
Never	92	24.0
1-2 times	114	29.7
3-4 times	4	1.0
Everyday	174	45.3

### Family cohesion

**Fig. 3:**
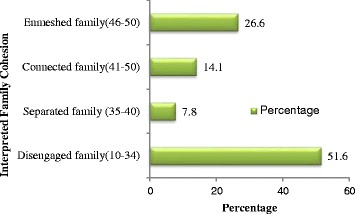
Percentage of the interpreted family cohesion from FACES-III

Family cohesion was measured using the cohesion scale from the Family Adaptability and Cohesion Evaluation Scale III (FACES-III). The majority of the respondents were from a disengaged family (i.e. extreme low cohesion) with an occurrence of 51.6 %. 26.6 % came from an enmeshed family, 14.1 % from a connected family and 7.8 % from a separated family (see Fig. 3). The mean value and standard deviation of the family cohesion score (FACES-III cohesion score) was 31.9 ± 12.7 showing an average perceived family cohesion.

### Socio-demographic variables and BMI

A strong statistically significant relationship (*P* <0.05) between BMI and the following socio-demographic variables (age group, ethnic group, demographic area, educational background, occupation and family history) was recorded. However, no significance was recorded between BMI with respect to the following demographic variables; sex (*P* = 0.554), marital status (*P* = 0.913), income group (*P* = 0.119), number of persons in family (*P* = 0.104) and type of diabetes (*P* = 0.973).

### Socio-demographic variables and number of meals per day

A strong significant relationship between age group (*P* = 0.000), occupation (*P* = 0.000), income group (*P* = 0.000) and type of diabetes (*P* = 0.001) with respect to number of meals per day was recorded. However no statistically significant relationship was noted for sex (*P* = 0.367), ethnic group (*P* = 0.054), demographic area (*P* = 0.060), marital status (*P* = 0.099), educational background (*P* = 0.126), number of persons in family (*P* = 0.347) and family history (*P* = 0.562) with respect to number of meals per day.

### Socio-demographic variables and skipping meals

A strong statistically significant relationship between age group (*P* = 0.000) and educational background (*P* = 0.000) with respect to skipping meals was recorded. However, no statistically significant relationship was noted for sex (*P* = 0.612), ethnic group (*P* = 0.078), demographic area (*P* = 0.341), marital status (*P* = 0.893), occupation (*P* = 0.073), income group (*P* = 0.674), type of diabetes (*P* = 0.075) and family history (*P* = 0.088) with respect to skipping meals.

### Socio-demographic variables and glycaemic index

A strong statistically significant relationship between age group (*P* = 0.001), occupation (*P* = 0.000), type of diabetes (*P* = 0.001) and family history (*P* = 0.008) with respect to glycaemic index was recorded. However no statistically significant relationship was found between sex (*P* = 0.454), ethnic group (*P* = 0.093), demographic area (*P* = 0.212), marital status (*P* = 0.562), educational background (*P* = 0.229), income group (*P* = 0.208), number of persons in family (*P* = 0.520) with respect to glycaemic index.

### Socio-demographic variables and average number of servings per week

A significantly strong relationship between ethnic group and chicken consumption (*P* = 0.001), beef (*P* = 0.000) and fish (*P* = 0.001) was recorded. Moreover, statistically strong relationship was found between demographic area and chicken (*P* = 0.000) and lamb (*P* = 0.000) consumption. Strong relationships were also found between income group and lamb (*P* = 0.001), brown bread (*P* = 0.001) and burger consumption (*P* = 0.001). Interestingly, the results also revealed strong statistically significant relationship between age group and burger (*P* = 0.000), chips (*P* = 0.000), pizza (*P* = 0.020), briani (*P* = 0.009) and fried noodles/rice consumption (*P* = 0.046). Strong relationships were equally found between educational background and burger (*P* = 0.000), chips (*P* = 0.000) and pizza consumption (*P* = 0.000). However there was no statistically significant relationship between the socio-demographic variables and the other remaining food items.

### Association between family meal frequency and family cohesion score

Table 6 shows the association between family meal frequency and family cohesion score. A very strong positive correlation between “eat breakfast together” (*r* = 0.850), “eat lunch together” (*r* = 0.895) and “eat dinner together” (*r* = 0.827) with respect to family cohesion score was recorded.

**Table 6:** Association between family meal frequency and family cohesion score

Family meal frequency	Family cohesion score (*r*)
Eat breakfast together	0.850**
Eat lunch together	0.895**
Eat dinner together	0.827**

**Correlation is significant at the 0.01 level

### Association between average number of servings per week of food items (individual dietary intake) and family cohesion score

Strong negative correlation between lamb/mutton (*r* = −0.631), white rice (*r* = −0.610), whole egg fried (*r* = −0.644), gateaux-piments (*r* = −0.674), burger (*r* = −0.657), chips (*r* = −0.669), briani (*r* = −0.671) and fried noodles/rice (*r* = −0.677) consumption with respect to perceived family cohesion score was recorded. Moreover, strong positive association was found between egg without yolk (*r* = 0.659), dried fruits (*r* = 0.731), low-fat milk (*r* = 0.682), cheese (*r* = 0.611), yogurt (*r* = 0.671), light butter (*r* = 0.627) with respect to perceived family cohesion score. Interestingly, very strong correlations were found between fish (*r* = 0.839), raw vegetables (*r* = 0.804), fresh fruits (*r* = 0.807) and nuts (*r* = 0.877) consumption with respect to family cohesion scores.

### Association between family meal frequency and individual dietary intake

For those who had breakfast together, there was a strong negative association ranging from −0.643 to – 0.716 with respect to family meal frequency. It was also noted that there was a strong positive correlation between raw vegetables ranging from 0656 to 0.788 with respect to family meal frequency. In addition, results equally demonstrated a very strong positive association between fish (*r* = 0.863) and nuts (*r* = 0.893) consumption with respect to family meal frequency. For those who ate lunch together, there was a strong negative association between lamb/mutton (*r* = −0.622), white rice (*r* = −0.658), white bread (*r* = −0.681), whole egg fried (*r* = −0.633), gateaux-piments (*r* = −0.691), burger (*r* = −0.666), chips (*r* = −0.676), briani (*r* = −0.681) and fried noodles/rice (*r* = −0.694) consumption with respect to family meal frequency. There was a statistically strong positive correlation between egg without yolk (*r* = 0.721), dried fruits (*r* = 0.709), low-fat milk (*r* = 0.652), yogurt (*r* = 0.681) and light butter (*r* = 0.658) consumption with respect to family meal frequency. It was also seen that there was a very strong positive association between fish (*r =* 0.873), raw vegetables (*r* = 0.875), fresh fruits (*r* = 0.863) and nuts (*r* = 0.919) consumption with respect to family meal frequency. For those diabetic participants who had dinner with their family, a strong negative correlation was found ranging from −0.612 to −0.795.

### Regression Analysis

Regression analysis was carried out only with the strong correlation values because the higher the correlation, the closer the points on the scatter plot will be on the regression line and the more confident one can be about the prediction.

### Relationship between family meal frequency and individual dietary intake (X➙Y)

Regression coefficients (*β*) and their standard error (SE) for the hierarchical linear regression models regressing average number of servings of the targeted food items (individual dietary intake) on family meal frequency in the three categories (“eat breakfast together”, “eat lunch together” and “eat dinner together”) are displayed in Table 7.

**Table 7:** Regression coefficients for average food servings per week regressed on family meal frequency

Dependent Variable(Y) Average servings/week	Independent Variable (X)					
	Eat breakfast together		Eat lunch together		Eat dinner together	
	β^a^±SE^b^	y-intercept	β^a^±SE^b^	y-intercept	β^a^±SE^b^	y-intercept
Lamb/mutton	−2.16 ± 0.120*	8.47	−2.12 ± 0.137*	7.93	−2.19 ± 0.147*	9.29
Fish	1.47 ± 0.044*	−0.88	1.59 ± 0.046*	−0.83	1.44 ± 0.066*	−1.29
Rice(white)	−1.86 ± 0.121*	10.6	−2.12 ± 0.124*	10.7	−2.08 ± 0.138*	11.8
Bread(white)	−1.99 ± 0.114*	10.9	−2.17 ± 0.120*	10.9	-	-
Whole egg fried	−2.01 ± 0.122*	8.14	−2.11 ± 0.132*	7.95	-	-
Egg without yolk	3.06 ± 0.122*	−1.06	0.84 ± 0.041*	−0.64	-	-
Raw vegetables	1.11 ± 0.045*	−0.77	3.64 ± 0.103*	−1.65	3.04 ± 0.163*	−2.07
Dried fruits	2.91 ± 0.120*	−0.51	1.08 ± 0.055*	−0.47	1.04 ± 0.062*	−0.98
Fresh fruits	1.61 ± 0.095*	0.03	3.45 ± 0.103*	−1.04	2.93 ± 0.156*	−1.58
Low-fat milk	1.25 ± 0.057*	−0.53	1.71 ± 0.102*	0.14	-	-
Cheese	1.39 ± 0.073*	−0.02	-	-	1.16 ± 0.076*	−0.75
Yogurt	−1.01 ± 0.061*	3.98	1.45 ± 0.080*	0.14	-	-
Nuts	1.91 ± 0.049*	−1.79	2.09 ± 0.046*	−1.80	1.93 ± 0.074*	−2.52
Gateaux-piments	−1.06 ± 0.053*	4.29	−1.09 ± 0.059*	4.14	−1.23 ± 0.059*	5.09
Light butter	0.96 ± 0.043*	−0.23	0.89 ± 0.052*	0.09	0.90 ± 0.057*	−0.42
Burgers	−1.68 ± 0.084*	6.61	−1.67 ± 0.096*	6.25	−2.06 ± 0.086*	8.22
Chips	−1.71 ± 0.082*	6.73	−1.69 ± 0.094*	6.33	−2.07 ± 0.084*	8.28
Briani	−1.59 ± 0.090*	6.56	−1.73 ± 0.095*	6.51	−2.14 ± 0.084*	8.56
Fried noodles/rice	−1.63 ± 0.080*	6.64	−1.76 ± 0.093*	6.57	−2.16 ± 0.081*	8.63

Note: all regression coefficients were adjusted for participant age and sex

*Significance is indicated with *P*-value < 0.05

^a^Regression coefficient. ^b^Standard error

### Relationship between family meal frequency and family cohesion (X➙M)

Regression coefficients (*β*) and their standard error (SE) for the hierarchical linear regression models regressing family cohesion scores on family meal frequency in the three categories (“eat breakfast together”, “eat lunch together” and “eat dinner together”) was carried out. The family cohesion score demonstrated strong positive associations when regressed on family meal frequency (*p* <0.005).

### Relationship between family cohesion and individual dietary intake (M➙Y)

Regression coefficients (*β*) and standard error (SE) for hierarchical linear regression models regressing average number of servings of the targeted food items on family cohesion scores was carried out. Significant positive associations for family cohesion scores and fish (*β* = 0.161), egg without yolk (*β* = 0.081), raw vegetables (*β* = 0.352), dried fruits (*β* = 0.117), fresh fruits (*β* = 0.340), low-fat milk (*β* = 0.189), cheese (*β* = 0.115), yogurt (*β* = 0.150), nuts (*β* = 0.211) and light butter (*β* = 0.090) were observed.

Significant negative associations were found for family cohesion scores and lamb/mutton (*β* = −0.227), white rice (*β* = −0.207), whole egg fried (*β* = −0.226), burger (*β* = −0.174), chips (*β* = −0.176), briani (*β* = −0.180) and fried noodles/rice (*β* = −0.180).

### Mediation Analysis (X➙M➙Y)

Table 8 shows the regression coefficients (*β*) and standard error (SE) for hierarchical linear regression models regressing average number of servings of the targeted food items on family cohesion scores, after controlling for frequency of family meals. It can be deduced that significant positive associations continued to be observed for family cohesion scores and intake of fish, egg without yolk, raw vegetables, dried fruits, fresh fruits, low-fat milk, cheese, yogurt and nuts after controlling for frequency of family meals, that is, “eat breakfast together”, “eat lunch together” and “eat dinner together”.

**Table 8:** Regression coefficients regressing average number of number of servings (individual dietary intake) on family cohesion scores and family meal frequency

Dependent Variable(Y)- Average number of servings/week	Independent Variable					
	Eat breakfast together		Eat lunch together		Eat dinner together	
	β^a^±SE^b^	y-intercept	β^a^±SE^b^	y-intercept	β^a^±SE^b^	y-intercept
Lamb/mutton	−0.07 ± 0.025*	10.4	−0.13 ± 0.032*	10.3	−0.13 ± 0.026*	12.2
	−1.64 ± 0.226*	10.4	−1.00 ± 0.300*	10.3	−1.07 ± 0.266*	12.2
Fish	0.07 ± 0.008*	−2.58	0.06 ± 0.010*	−1.99	0.12 ± 0.010*	−3.43
	0.94 ± 0.075*	−2.58	1.08 ± 0.098*	−1.99	0.45 ± 0.098*	−3.43
Rice(white)	−0.09 ± 0.024*	14.9	−0.04 ± 0.028	13.8	−0.05 ± 0.023*	17.1
	−1.19 ± 0.217*	14.9	−1.73 ± 0.271*	13.8	−1.77 ± 0.237*	17.1
Whole egg fried	−0.12 ± 0.025*	11.2	−0.14 ± 0.030*	10.8	−0.17 ± 0.026*	12.3
	−1.11 ± 0.226*	11.2	−0.96 ± 0.290*	10.8	−0.65 ± 0.260*	12.3
Egg without yolk	0.07 ± 0.008*	−2.92	0.01 ± 0.009*	−1.99	0.09 ± 0.008*	−2.49
	0.11 ± 0.075	−2.62	0.73 ± 0.087*	−1.99	−0.19 ± 0.840*	−2.49
Raw vegetables	0.20 ± 0.023*	−6.16	0.05 ± 0.023*	−3.95	0.28 ± 0.024*	−7.62
	1.56 ± 0.203*	−6.16	3.15 ± 0.223*	-.3.95	0.81 ± 0.242*	−7.62
Dried fruits	0.04 ± 0.009*	−1.85	0.07 ± 0.012*	−1.86	0.09 ± 0.010*	−2.54
	0.85 ± 0.084*	−1.85	0.41 ± 0.117*	−1.86	0.33 ± 0.105*	−2.54
Fresh fruits	0.21 ± 0.022*	--6.53	0.08 ± 0.023*	−4.64	0.26 ± 0.022*	−7.99
	1.38 ± 0.191*	−6.53	2.71 ± 0.217*	−4.64	0.91 ± 0.22*	−7.99
Low-fat milk	0.11 ± 0.017*	−5.29	0.15 ± 0.021*	−5.26	0.13 ± 0.017*	−6.19
	0.81 ± 0.154*	−5.29	0.43 ± 0.200*	−5.26	0.57 ± 0.177*	−6.19
Cheese	0.01 ± 0.012*	−0.15	0.08 ± 0.017*	−0.49	0.06 ± 0.014*	−1.36
	1.35 ± 0.109*	−0.15	0.27 ± 0.162	−0.49	0.64 ± 0.141*	−1.36
Yogurt	0.05 ± 0.014*	−3.74	0.07 ± 0.017*	−3.46	0.09 ± 0.015*	−4.77
	1.01 ± 0.125*	−3.74	0.79 ± 0.166*	−3.46	0.62 ± 0.149*	−4.77
Nuts	0.09 ± 0.009*	−3.51	0.06 ± 0.010*	−2.63	0.15 ± 0.010*	−4.70
	1.16 ± 0.079*	−3.51	1.52 ± 0.098*	−2.63	0.72 ± 0.105*	−4.70
Gateaux-piments	−0.04 ± 0.011*	5.56	−0.04 ± 0.014*	5.27	−0.02 ± 0.010*	6.94
	−0.78 ± 0.099*	5.56	−0.69 ± 0.130*	5.27	−1.09 ± 0.107*	6.94
Light butter	0.01 ± 0.009	−0.61	0.02 ± 0.012*	−0.46	0.04 ± 0.010*	−1.62
	1.02 ± 0.081*	−0.61	0.66 ± 0.117*	−0.46	0.59 ± 0.105*	−1.62
Burgers	−0.04 ± 0.018*	7.26	−0.07 ± 0.022*	6.90	0.02 ± 0.016*	9.83
	−1.31 ± 0.159*	7.26	−1.02 ± 0.212*	6.90	−2.13 ± 0.161*	9.83
Chips	−0.04 ± 0.017*	7.59	−0.08 ± 0.022*	7.31	0.00 ± 0.015	10.2
	−1.38 ± 0.155*	7.59	−0.98 ± 0.209*	7.31	−2.13 ± 0.157*	10.2
Briani	−0.10 ± 0.019*	6.74	−0.08 ± 0.022*	6.12	−0.01 ± 0.016	9.07
	−0.83 ± 0.165*	6.74	−1.07 ± 0.208*	6.12	−2.10 ± 0.159*	9.07
Fried noodles/rice	−0.08 ± 0.018*	7.57	−0.07 ± 0.022*	6.95	0.01 ± 0.015	10.1
	−0.95 ± 0.163*	7.57	−1.14 ± 0.206*	6.75	−2.29 ± 0.151*	10.1

Note: all regression coefficients were adjusted for participant age and sex

*Significance is indicated with *P*-value < 0.05, ^a^Regression coefficient, ^b^Standard error

Family Cohesion Score adjusted for family meal frequency (X); Family meal frequency adjusted for family cohesion scores (M)

These findings indicate a 52.6 % reduction for the association of family meal frequency and individual dietary intake of the targeted food items as mediated by family cohesion. The calculation was done by subtracting the adjusted regression coefficient for family meal frequency from its unadjusted counterpart and then dividing by the unadjusted regression coefficient for frequency of family meals. The percentage reduction obtained clearly indicated a more or less complete mediation (>50 %) for the association between family meal frequency and individual dietary intake as mediated by family cohesion. Sobel’s test, carried out using SPSS version 16.0, confirmed the trend towards complete mediation by family cohesion for the relationship between family meal frequency and individual dietary intake as indicated with z = 15.4 and *P* = 0.000.

## Discussion

The main aim of this study was to examine the relationship between family meal frequency and individual dietary intake among diabetic patients in Mauritius. In addition to the primary aim, it was also hypothesised that family cohesion may be a plausible mediator of this relationship. To our best of knowledge, no such studies have evaluated the association between family meal frequency, individual dietary intake and family cohesion among diabetic patients in Mauritius. It was hypothesised that family meal frequency would be positively associated with healthy food items intake (chicken, fish, liver/organ meat, pulses, brown rice, brown bread, boiled egg, egg without yolk, tofu, soya, vegetables, fruits, nuts, low-fat milk, cheese, yogurt, light butter, fresh juice, water and artificial sweeteners) and negatively associated with less healthy food items intake (lamb/mutton, beef, white rice, white bread, dholl-puri/farata, full-fat milk, oily and sugary snacks, ghee/saturated fats, soft drinks and fast foods). Moreover, an endeavour has been made to assess a series of socio-demographic variables including sex, age group, ethnic group, demographic area, marital status, educational background, occupation, income group, number of family members, type of diabetes and family history of diabetes in relation to glycaemic index, skipping meals, number of meals per day, intake of targeted food items and BMI.

This study has shown that 40.9 %, 28.1 % and 45.3 % of the diabetic patients had breakfast together, eat lunch and dinner together respectively. Mellin et al. [20] have found that 57 % of families have regular and frequent family meals which were more or less in line with our findings. Additionally, results have also suggested that greater frequency of family meals was significantly associated with greater average servings of fish, raw vegetables, dried fruits, fresh fruits, low-fat milk, cheese, yogurt, nuts and light butter. Greater family meal frequency demonstrated a trend towards lower average servings of lamb/mutton, white rice, white bread, whole egg fried, chocolates, gateaux-piments, burgers, chips, briani and fried noodles/rice. Previous studies by Andaya et al. [3] have shown that children consuming breakfast, lunch and dinner with their family at least 4 days per week had a percentage intake of 84 %, 85 % and 80 % of fruits and vegetables respectively. Moreover, the study supported our findings by indicating only a 40 %, 44 % and 43 % of soda and chips consumption when children ate breakfast, lunch and dinner with their families respectively. Positive associations between regular family meals and daily intake of vegetables, calcium-rich food, fibre, calcium, magnesium, potassium, zinc, folate, vitamin A and B_6_ and negative associations with fast- food intake were also found by Burgess-Champoux et al. [5]. Another study by Larson et al. [17], which was in line with our results, reported that eating dinner was significantly associated with higher dietary intakes of vegetables and fruits. They equally reported that young adults enjoyed and valued eating with other family members. Fulkerson et al. [10] have demonstrated that family dinner frequency was positively associated with fruit consumption. Therefore our present findings were somewhat consistent with previous literature.

To our best of knowledge, no such studies have examined the association between family cohesion, family meal frequency and dietary intake among diabetic patients. Studies previously done on family cohesion are relatively few and none of them have really investigated on dietary consumption. Combrey et al. [10] have shown that adolescent reported overeating was associated with lower scores of family cohesion and adaptability. Significant negative associations were indicated by Patton et al. [26] between the dietary adherence of children and two dimensions of family function (task accomplishment and behavioural control). Meunier et al. [21] showed that the perception of family cohesion by mothers was a predictor of the number of severe hypoglycaemic events through hierarchical regression analyses. Most studies were conducted on family support and the diabetic management of patients and not really family cohesion. Skinner and Hampson [29] have found that the general family support was s significant predictor (β = 0.37, *p* < 0.05) of the efficacy of treatment in controlling diabetes. According to Geffken et al. [11], higher child perceptions of parental warmth and caring were associated with decreased odds of experiencing a diabetic ketoacidosis. In addition, Rosenberg and Shields [27] found that greater maternal perceptions of more secure attachment of adolescents was associated with better glycaemic control.

However, only one study investigated the association between family cohesion and individual dietary intake. Welsh et al. [32] have shown significant negative associations between family cohesion and sweets intake. These findings were somewhat in line with our results that suggested significant negative associations between family cohesion scores and chocolates intake as well as lamb/mutton, white rice, white bread, whole egg fried, gateaux-piments, burgers, chips, briani and fried noodles/rice consumption. Furthermore, our results equally demonstrated significant positive associations between family cohesion and fish, raw vegetables, dried fruits, fresh fruits, low-fat milk, cheese, yogurt, nuts and light butter consumption. Welsh et al. [32] also demonstrated a significant positive association between family meal frequency and family cohesion score which was consistent with our findings as significant positive associations were equally obtained.

Welsh et al. [32] have also hypothesised that family cohesion may be a potential predictor of the association between family meal frequency and individual dietary intake. Although significant associations were demonstrated between family cohesion and dietary intake of sweets in the study, the authors failed to show statistically significant associations between the two variables after adjusting for family meal frequency using hierarchical linear regression models. These findings, therefore, were unlikely to predict any mediation association. Welsh et al. [32] equally obtained a 21 % reduction for the association of family meals and sweets consumption as mediated by family cohesion and the Sobel’s test suggested a trend towards a partial mediation. With this interpretation of data, there was no significant evidence for the mediation of family meal frequency and individual dietary intake by family cohesion. In contrast to the results obtained, our results demonstrated strong correlation values between family cohesion score and individual dietary intake after adjusting for family meal frequency and vice versa. A 52.6 % reduction was obtained for the association of family meal frequency and individual dietary intake as mediated by family cohesion score which indicated more or less complete mediation and the findings were confirmed with the Sobel’s test. The reason for these differences in findings as compared to the previous study may be due to the smaller sample size used. The different cultural belongings of the Mauritian population may equally play a role in the research study being investigated.

This study has also taken into account the socio-demographic variables in relation to BMI, skipping meals, glycaemic index, number of meals per day and the average servings of the targeted food items. Our findings indicated that most of the diabetic patients actually consumed three meals per day (52.6 %) instead of the recommended number of meals, that is, five meals per day (28.9 %). It was also noted that 60.9 % of the participants did not skip meals and only 39.3 % actually did skip meals. Significant differences were found between age group and skipping meals as well as for education and skipping meals. A decline in breakfast consumption by mostly adolescents has been documented in the United Sates [1]. The reasons for the relationship between skipping meals and age group may be due to the fact that children and adolescents are usually engrossed in irregular activities. Home works, lack of time or any other social gathering may usually contribute to the skipping of meals. Estima et al. [9] have found that breakfast skipping was more common among girls (12.4 %) rather than boys (4.5 %). These findings were not in line with our results as no significant differences were noted between gender and skipping meals. It has been reported by Gross et al. [12] that urban students were twice more likely to skip breakfast meals compared to rural and suburban students. In contrast, the present study did not suggest any significant differences between demographic area and skipping meals.

Statistically significant relationships were noted for age group, ethnic group, demographic area, education, occupation and family history in relation to BMI. O’Neil et al. [25] observed a significant trend in age groups for increased consumption of whole grains with lower BMI. Various studies have demonstrated significant relationships between BMI and gender. Lynch et al. [19] have found that BMI z-scores were significantly higher for boys and girls. Srdić et al. [30] and Morimoto et al. [23] have found strong correlation between BMI and percentage body fat among girls comparing to boys. Significant differences between males and females in the BMI categories were equally indicated by James [15]. However these findings were not consistent with the present study as no relationship between gender and BMI was noted.

Interestingly, our results have shown that 97.4 % of the diabetic patients did not have any knowledge about glycaemic index and only 2.6 % were aware of the meaning of glycaemic index. Many studies have shown the importance of consuming a low- glycaemic index meal in order to control blood glucose levels and unfortunately in the present study most of the respondents were unaware of the situation. Yusof et al. [34] has found that a low-glycaemic index diet was associated with significant changes in fructosamine level and plasma glucose in Asian patients with Type II diabetes. Jimérez-Cruz et al. [16] have indicated that low-glycaemic index diets have the potential of increasing satiety and decreasing the incidence of diabetes and produce better glycaemic control. Age, occupation, type of diabetes and family history of diabetes were all significantly related to glycaemic index. A proper explanation is necessary to enable the patients to adopt healthy behaviours and to make sensible food choices. Furthermore, adolescents are not exposed to the concept of glycaemic index in schools which means a lower knowledge of the importance of consuming low-glycaemic foods.

Income was a determining variable in the consumption of certain food items. There were statistically significant relationships between income group and consumption of lamb/mutton, brown bread and burgers. Deshmukh-Taskar et al. [7] have found that higher incomes (> $45, 000) had lower consumption of burgers or sandwiches. Burnier et al. [6] have found significant associations between high energy intake and income group. The reason for the relationship between lamb/mutton and brown bread in relation to income in Mauritius may be due to the fact that these food products are relatively expensive and not everybody has the means of affording them. As far as burgers are concerned, they are relatively cheap food items on sale in Mauritius and many individuals are able to readily consume them. Ding et al. [8] have demonstrated that family income was associated with availability of more-healthful food in the home. Furthermore, Mushi-Brunt et al. [24] found no significant differences in fruits and vegetables intake by income status. These findings were much in line with our results which suggested no relationships between income group and fruit and vegetable consumption.

The specificity of the study is the use of a validated method, Family Adaptability and Cohesion Evaluation Scale III (FACES-III) by Baron and Kenny [4], to measure family cohesion which provides a more objective assessment of the present study. Our main findings suggest a strong association between family meal frequency and individual dietary intake of Mauritian diabetic patients and confirm our hypothesis that family cohesion is a plausible mediator of this relationship. The results obtained suggest that not only adolescents but even adults’ dietary behaviours are influenced by family characteristics and functioning. Hence, there is a clear demand that both parents and their respective families should benefit from intense behavioural-based interventions that require family members to consider reviewing rules, role modelling, establish limits at mealtimes and adopt positive attitudes when eating together.

Even though our findings precisely demonstrate a relationship between family meal frequency and individual dietary intake, future studies can formulate more effective evaluation tools that accurately measure the frequency of family meals and the quality of meals being served at home. Since not many studies have been conducted on these three parameters, several questions ultimately stem from the results of this study indicating a need for immediate and positive intervention within the field of family meal environment and connectedness.

## Conclusions

A decline in family meals across generations has been observed and this trend may not necessarily be due to the fact that family members do not enjoy eating together. Busy work schedules, children’s activities after school, television viewing and new methods of processing and distributing foods such as pre-packaged meals may all be contributing factors to the demise of family members sitting together for a meal. Previous research has investigated and demonstrated the numerous benefits of family meals including greater consumption of healthy foods, adolescent’s health status, glycaemic control and lower fasting total cholesterol. Our findings have shown that greater frequency of family meals was significantly associated with greater consumption of healthy foods such as fish, raw vegetables, dried fruits, fresh fruits, low-fat milk, cheese, yogurt, nuts and light butter, that is, better individual dietary intake. Thus it can be recommended that specific nutrition intervention programs encouraging the frequency of more family meals need to be targeted to the diabetic population as well as their respective families. Furthermore, the measurement of family cohesion as a plausible mediator of this relationship adds to the uniqueness of the study. We recorded a 52.6 % reduction for the association of family meal frequency and individual dietary intake as mediated by family cohesion which indicated a more or less complete mediation. Henceforth, it can be concluded that health professionals should take into consideration the connectedness of family members in order to devise innovative nutrition intervention strategies. Therapeutic and psychological interventions, involving family members, can be adopted for the management of diabetes. However, future research in this area is needed to assess other mediators such as parents’ rules at home, time spent in food preparation and availability of home-made meals.
